# An advanced workflow for single-particle imaging with the limited data at an X-ray free-electron laser

**DOI:** 10.1107/S2052252520012798

**Published:** 2020-10-15

**Authors:** Dameli Assalauova, Young Yong Kim, Sergey Bobkov, Ruslan Khubbutdinov, Max Rose, Roberto Alvarez, Jakob Andreasson, Eugeniu Balaur, Alice Contreras, Hasan DeMirci, Luca Gelisio, Janos Hajdu, Mark S. Hunter, Ruslan P. Kurta, Haoyuan Li, Matthew McFadden, Reza Nazari, Peter Schwander, Anton Teslyuk, Peter Walter, P. Lourdu Xavier, Chun Hong Yoon, Sahba Zaare, Viacheslav A. Ilyin, Richard A. Kirian, Brenda G. Hogue, Andrew Aquila, Ivan A. Vartanyants

**Affiliations:** a Deutsches Elektronen-Synchrotron DESY, Notkestraße 85, Hamburg, D-22607, Germany; b National Research Center ‘Kurchatov Institute’, Akademika Kurchatova pl. 1, Moscow, 123182 Russian Federation; c National Research Nuclear University MEPhI (Moscow Engineering Physics Institute), Kashirskoe sh. 31, Moscow, 115409, Russian Federation; d Department of Physics, Arizona State University, Tempe, Arizona AZ 85287, USA; e School of Mathematics and Statistical Sciences, Arizona State University, Tempe, Arizona AZ 85287, USA; f Institute of Physics, ELI Beamlines, Academy of Sciences of the Czech Republic, Prague, CZ-18221, Czech Republic; g Australian Research Council Centre of Excellence in Advanced Molecular Imaging, Department of Chemistry and Physics, La Trobe Institute for Molecular Science (LIMS), La Trobe University, Melbourne, Victoria 3086, Australia; h School of Life Sciences, Arizona State University, Tempe, Arizona AZ 85287, USA; i Biodesign Institute Center for Immunotherapy, Vaccines and Virotherapy, Arizona State University, Tempe, Arizona AZ 85287, USA; j Stanford PULSE Institute, SLAC National Accelerator Laboratory, 2575 Sand Hill Road, Menlo Park, CA 94025, USA; k Department of Molecular Biology and Genetics, Koc University, Istanbul, 34450, Turkey; l Center for Free Electron Laser Science (CFEL), DESY, Notkestraße 85, Hamburg, D-22607, Germany; m Laboratory of Molecular Biophysics, Department of Cell and Molecular Biology, Uppsala University, Husargatan 3, Uppsala, SE-75124, Sweden; n SLAC National Accelerator Laboratory, 2575 Sand Hill Road, Menlo Park, CA 94025, USA; o European XFEL, Holzkoppel 4, Schenefeld, D-22869, Germany; p Physics Department, Stanford University, 450 Jane Stanford Way, Stanford, CA 94305-2004, USA; q School for Engineering of Matter, Transport and Energy, Arizona State University, Tempe, AZ 85287, USA; r University of Wisconsin Milwaukee, Milwaukee WI 53211, USA; s Moscow Institute of Physics and Technology, Moscow, 141700, Russian Federation; t Max-Planck Institute for the Structure and Dynamics of Matter, Luruper Chaussee 149, Hamburg, D-22761, Germany; u Biodesign Institute, Center for Applied Structural Discovery, Arizona State University, Tempe, AZ 85287, USA

**Keywords:** single-particle imaging, three-dimensional virus reconstruction, bacteriophage PR772, XFELs

## Abstract

An improved analysis for single-particle imaging (SPI) experiments, using the limited data, is presented here. Results are based on a study of bacteriophage PR772 performed at the Atomic, Molecular and Optical Science instrument at the Linac Coherent Light Source as part of the SPI initiative. Existing methods were modified to cope with the shortcomings of the experimental data: inaccessibility of information from half of the detector and a small fraction of single hits. The general SPI analysis workflow was upgraded with the expectation-maximization based classification of diffraction patterns and mode decomposition on the final virus-structure determination step. The presented processing pipeline allowed us to determine the 3D structure of bacteriophage PR772 without symmetry constraints with a spatial resolution of 6.9 nm. The obtained resolution was limited by the scattering intensity during the experiment and the relatively small number of single hits.

## Abstract 2

The 3D structure of bacteriophage PR772 was determined from a single-particle imaging experiment at the Linac Coherent Light Source, using the limited number of data, with a spatial resolution of 6.9 nm.

## 1. Introduction   

The recent worldwide outbreak of the COVID-19 pandemic has indicated urgency for the development of complementary imaging techniques for the study of virus structures at high resolution. Currently, cryogenic electron microscopy (cryo-EM) outperforms other methods of analysis of single virus particles, although to determine the structure one needs to freeze the samples at cryogenic temperatures. Understanding the nature of the same viruses at room temperature as well as in aerosols may provide information on the actual size distribution of virus particles in aerosols, a factor that may affect infection of humans by these viruses.

As suggested about two decades ago, intense femtosecond pulses from X-ray free-electron lasers (XFELs) may outrun conventional radiation damage, found at synchrotron X-ray sources, or the Coulomb explosion, found at the intensities of XFELs. They therefore allow, in principle, atomic resolution structure determination of isolated macromolecules in their native environment at room temperatures (Neutze *et al.*, 2000 ▸). After the start of the first hard X-ray FELs (Emma *et al.*, 2010 ▸; Ishikawa *et al.*, 2012 ▸), it became clear that atomic resolution could be achieved with intense X-ray pulses from an XFEL on micro- and nano-crystals of proteins, leading to the serial femtosecond crystallography (SFX) technique (Chapman *et al.*, 2011 ▸; Aquila *et al.*, 2012 ▸; Boutet *et al.*, 2012 ▸). However, progress toward high-resolution 3D electron-density images of non-crystalline biological particles using the single-particle imaging (SPI) approach has been slow compared with SFX (Bogan *et al.*, 2008 ▸; Seibert *et al.*, 2011 ▸; Hantke *et al.*, 2014 ▸; van der Schot *et al.*, 2015 ▸; Ekeberg *et al.*, 2015 ▸).

There are several reasons for the lower resolution achieved in SPI experiments in comparison with the SFX technique. The most important include: lack of crystalline periodicity to amplify the signal, weak single-particle signal from non-periodic nanoscale objects compared with instrumental background, limited number of usable data frames collected and heterogeneity of the samples. Radiation-damage processes initiated by X-ray photoionization may also play an important role at high resolution, but this limitation is minimized at XFEL sources by performing experiments in the ‘diffract and destroy’ mode (Chapman *et al.*, 2006 ▸). One may expect that in order to further enhance resolution, one could increase the power of XFEL pulses to boost the signal-to-noise ratio for bio-particles, such as single proteins or virus particles that scatter very weakly. Unfortunately, increased XFEL fluence strips electrons from atoms more efficiently and the scattered power from the bound electrons (which contains structural information) does not increase in proportion to the X-ray fluence (Lorenz *et al.*, 2012 ▸; Gorobtsov *et al.*, 2015 ▸). Decreasing the pulse length below 1 fs may help to outrun Auger decay, but further decrease of the pulse duration may not lead to the desired suppression of all ionization channels (Gorobtsov *et al.*, 2015 ▸).

Beside these fundamental limitations, there are a few other limiting factors. Background scattering originating from the beamline obscures the weak scattering signals from biological samples, and fluctuations of the beam position and intensity can cause additional variables that need to be accounted for in reconstruction algorithms. Moreover, single-particle sample delivery remains a challenging topic. In addition, despite strong development of detector technology [see, for example, Allahgholi *et al.* (2019 ▸)], the dynamic range of the present detectors is still not sufficient to capture a full diffraction pattern in a strong single shot. To tackle all these limitations of the SPI technique and to push the methodology further, a dedicated SPI consortium was formed at the Linac Coherent Light Source (LCLS) (Aquila *et al.*, 2015 ▸).

Several results were reported from the SPI consortium, covering both hard and soft X-ray experiments and focused primarily on well characterized viruses with sizes of a few tens of nanometres. Different analysis methods have been applied to virus structure determination [see, for example, Munke *et al.* (2016 ▸), Reddy *et al.* (2017 ▸), Kurta *et al.* (2017 ▸), Rose *et al.* (2018 ▸) and Shi *et al.* (2019 ▸)]. In the present work, we advance the previous studies performed on bacteriophage PR772 (Rose *et al.*, 2018 ▸) by extending the general analysis pipeline of the SPI data as first proposed by Gaffney & Chapman (2007) ▸. The main upgraded features include: a classification method based on the expectation-maximization (EM) algorithm, developed in cryo-EM (Dempster *et al.*, 1977 ▸), and mode decomposition for the final virus structure determination (Vartanyants & Singer, 2010 ▸; Thibault & Menzel, 2013 ▸). EM-based algorithms were first applied in SPI data analysis for 3D orientations recovery (Loh & Elser, 2009 ▸; Ayyer *et al.*, 2016 ▸). Below we present a detailed description of all the steps that allowed us to obtain the electron density of the PR772 virus with an improved resolution compared with the previous SPI studies on the same virus (Rose *et al.*, 2018 ▸).

## 2. Experiment   

The experiment was performed in the Atomic, Molecular and Optical Science (AMO) instrument (Bozek, 2009 ▸; Ferguson *et al.*, 2015 ▸; Osipov *et al.*, 2018 ▸) at the LCLS at SLAC National Accelerator Laboratory using the LAMP end station [see Li *et al.* (2020 ▸) for experimental details]. PR772 bacteriophage growth and purification were carried out essentially as previously described (Reddy *et al.*, 2017 ▸). Viruses in ammonium acetate volatile buffer were aerosolized in a helium environment using gas dynamic virtual nozzles (GDVN) that were 3D printed via 2 photon polymerization photo-lithography with a Nanoscribe Professional GT printer (Nazari *et al.*, 2020 ▸). Laboratory measurements showed reproducible jet diameters in the range of 0.5–2.0 µm (DePonte *et al.*, 2008 ▸; Weierstall *et al.*, 2012 ▸). The particles then passed through a differentially pumped skimmer for pressure reduction and were then injected/focused into the sample chamber using an aerodynamic lens injector (Benner *et al.*, 2008 ▸; Hantke *et al.*, 2014 ▸). The focused particle stream intersected the focused and pulsed XFEL beam.

**Figure 1:**
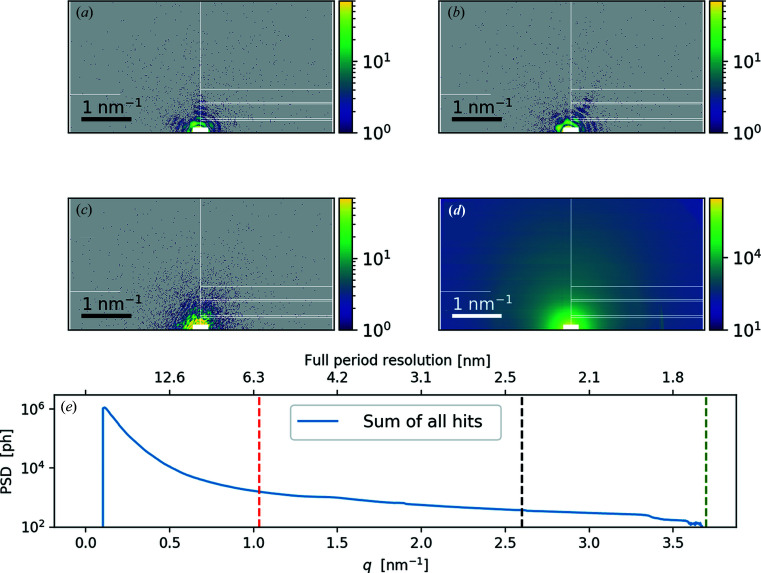
Examples of diffraction patterns from the SPI experiment. (*a*), (*b*) Diffraction patterns corresponding to a single PR772 virus hit by an XFEL beam. (*c*) A diffraction pattern corresponding to a non-single hit which was sorted out from the analysis at the classification step. (*d*) A sum of 1.9 × 10^5^ diffraction patterns identified as hits. White regions in the center of the diffraction patterns as well as white stripes correspond to a mask introduced to reduce artefacts owing to the signal exceeding the detector capabilities. The mask was the same before and after the move of the detector panel. (*e*) The PSD function of the scattered intensity for the sum of all diffraction patterns identified as hits collected in the experiment. The signal until the corner and edge of the detector is indicated by the green and black vertical dashed lines, respectively. For orientation determination the data until momentum-transfer values of 1 nm^−1^ (shown by the red dashed line) were used.

The LCLS had a repetition rate of 120 Hz, for this experiment the average pulse energy was ∼4 mJ, with a focal diameter of ∼1.5 µm and a photon energy of 1.7 keV (wavelength 0.729 nm). Diffraction patterns were recorded by a pnCCD detector (Strüder *et al.*, 2010 ▸) mounted at a 0.130 m distance from the interaction region. In the experiment, a silver behenate salt was used as a calibration agent to determine sample detector distance and panel position. The scattering signal was only recorded by one of the two detector panels (one panel was not operational during the experiment because of an electronic fault). The size of the working panel was 512 by 1024 pixels with a pixel size of 75 × 75 µm^2^, with the long edge closest to the interaction point. In the middle of the experiment (at run 205) the detector panel was moved 1 mm vertically up relative to the incoming X-ray beam to reduce background scattering. Our work is based on the data obtained from this panel covering a part of reciprocal space, as shown in Figs. 1 ▸(*a*)–1 ▸(*d*).

## 3. Initial classification steps   

SPI data analysis involves many subsequent steps leading to the 3D reconstructed particle structure from a large set of 2D diffraction patterns (Gaffney & Chapman, 2007 ▸). Improvements in the data-analysis pipeline at early stages can result in a significant enhancement in reconstruction quality. Therefore, several pre-processing methods were developed to avoid experimental artifacts on the collected diffraction patterns. The pre-processing stages include: hit finding, background correction, beam-position refinement and particle-size filtering.

The initial experimental dataset, as collected at the AMO instrument, consists of ∼1.2 × 10^7^ diffraction patterns (9 × 10^6^ patterns before and 3 × 10^6^ patterns after moving the detector panel) (see Li *et al.*, 2020 ▸). The hit finding was performed using the software *psocake* in the *psana* framework (Damiani *et al.*, 2016 ▸). As a result, 1.9 × 10^5^ diffraction patterns were identified as hits from the initial set of diffraction patterns (see Table 1 ▸) and the signal from these hits was converted to photon counts. Examples of selected diffraction patterns are shown in Fig. 1 ▸.

Visual inspection of the selected patterns revealed the presence of additional instrumental scattering near the center of the diffraction patterns [see Fig. S1(*a*) in the Supporting information]. The scattering was caused by the interaction of the tails of the FEL beam with the upstream apertures or from the sample injector. To subtract this additional signal, histograms of intensity for each pixel were analyzed (Figs. S1 and S2). It was assumed that for pixels with zero photon counts, the histogram of intensity distribution should have a Gaussian form and a mean value equal to zero. The mean value of this distribution was subtracted from each diffraction pattern. Subtraction of this additional scattering contribution was an important step prior to finding the beam center position (see the Supporting information for details).

**Figure 2:**
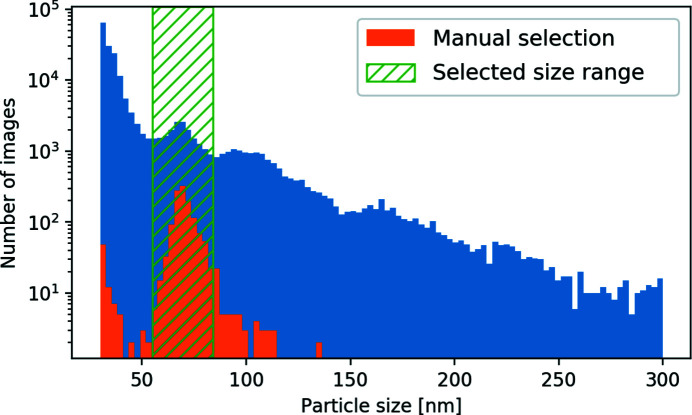
A particle-size histogram after the PSD-function filtering. The blue area corresponds to 1.8 × 10^5^ diffraction patterns, satisfying the fidelity score criterion FS > 1.05 (see the Supporting information for details). The orange area corresponds to manually selected single-hit diffraction patterns. A range of particle sizes from 55 to 84 nm (1.8 × 10^4^ diffraction patterns) was selected for the further SPI analysis (green dashed area).

The particle-size filtering was performed in two steps. First, the power spectral density (PSD) function, *i.e.* angular averaged intensity, of each diffraction pattern was fitted with the form factor of a solid sphere in a wide range of sizes from 30 to 300 nm. Next, a fidelity criterion was introduced to distinguish between successful and unsuccessful size findings (see the Supporting information for details). As an outcome of this analysis, the number of selected diffraction patterns was reduced from 1.9 × 10^5^ to 1.8 × 10^5^, which were used for the final particle-size filtering. A histogram for different particle sizes in the selected size range is shown in Fig. 2 ▸. An extended range of sizes observed in this figure corresponds to clusters of particles stuck together and the varying thickness of a hydration layer. One can also identify a peak in this histogram in the range from 55–84 nm. This size range agrees well with the expected virus particle size of ∼70 nm (Rose *et al.*, 2018 ▸; Reddy *et al.*, 2019 ▸). The considered range contains 1.8 × 10^4^ diffraction patterns, which were selected for further single-hit classification (see Table 1 ▸).

## 4. Single-hit diffraction patterns classification   

The key step of data selection in this work was single-hit classification. The angular X-ray cross-correlation analysis classification that was used in the previous work (Bobkov *et al.*, 2015 ▸; Rose *et al.*, 2018 ▸) was not as effective with the present experimental data owing to the absence of the scattering signal on one half of the detector and the low single-hit rate.

The single-hit classification approach was based on the EM algorithm (Dempster *et al.*, 1977 ▸; Loh & Elser, 2009 ▸; Ayyer *et al.*, 2016 ▸). This algorithm allows for unsupervised clustering of data when neither initial data assignments to clusters nor cluster parameters are known. The dataset is distributed into a pre-defined number of clusters and cluster parameters are retrieved automatically at the same time. Later, manual input is used to classify each of the cluster models, based on symmetry considerations or expected fringe patterns, in order to perform data selection. This algorithm is commonly used in cryo-EM for unsupervised single-particle clustering [see, for example, the software *RELION* (Scheres *et al.*, 2005 ▸)]. Low contrast and low signal-to-noise level are common problems for cryo-EM and SPI so we implemented in this work the original EM algorithm developed in cryo-EM for clustering of SPI data.

**Figure 3:**
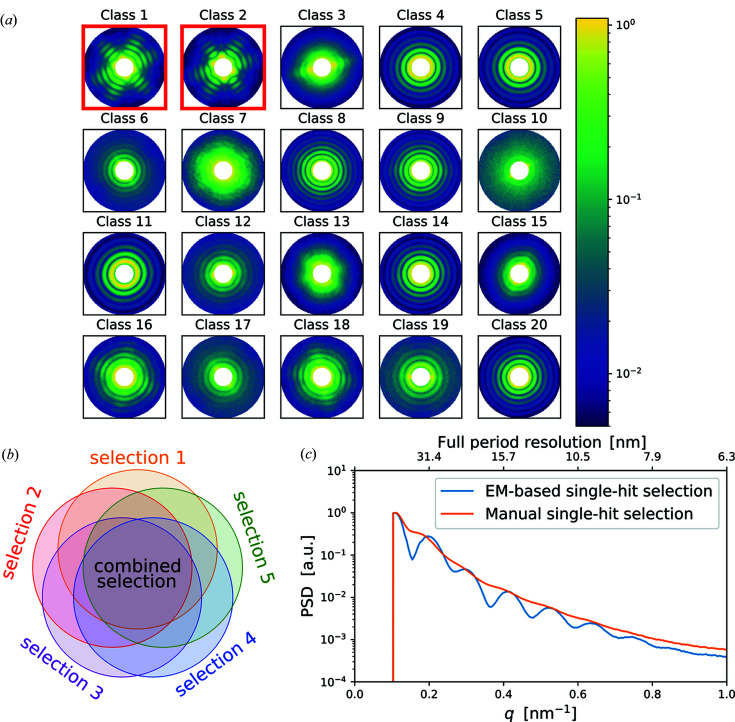
Classification of diffraction patterns by EM clustering. (*a*) Diffraction patterns are distributed into 20 classes according to their features. Classes 1 and 2 were selected as they clearly contain structural features of the investigated virus and its icosahedral shape. These two classes contain 1609 diffraction patterns. (*b*) EM-clustering was repeated five times, and intersecting selection with 1085 patterns was considered for further analysis. (*c*) Averaged PSD functions for EM-based single-hit selection containing 1085 patterns (blue line) and for manual selection containing 1393 patterns (orange line).

Unsupervised EM clustering is an iterative process; the algorithm starts from a random model for each class. At each iteration the probability for each diffraction pattern to belong to a certain class is calculated. To accommodate for random particle orientations in the SPI experiment, the cluster model is compared with the 2D diffraction pattern rotated in-plane. After probabilities are evaluated, a new cluster model is calculated by weighted averaging of patterns that belong to each cluster in each orientation. The weights are defined by computed probabilities. In fact, the algorithm imitates the expand–maximize–compress (EMC) algorithm (Loh & Elser, 2009 ▸) but, instead of rotation in 3D space, an in-plane rotation and cluster distribution are used (Loh, 2014 ▸). When the EM algorithm converges, the supervised class selection takes place. The cluster models that correspond to single hits of an investigated particle are selected manually by an expert. As an example, in one of the EM clustering we selected classes 1 and 2 as classes containing six high-contrast fringes that we attribute to scattering from a PR772 virus particle [see Fig. 3 ▸(*a*)].

To make classification more accurate we performed five independent EM clusterings starting from random cluster models. Each EM clustering produced slightly different results, which are summarized in Table 1 ▸. The intersection of all the results was considered as a stable single-hit selection [see Fig. 3 ▸(*b*)]. Finally, we ended with the dataset containing 1085 single-particle patterns (see Table 1 ▸). The PSD function of an average of all the selected diffraction patterns is shown in Fig. 3 ▸(*c*). The scattering signal from the virus particle extends up to the momentum-transfer value *q*_max_ = 1 nm^−1^ in reciprocal space, which corresponds to a resolution (2π/*q*_max_) of 6.3 nm in real space.

A manual search and selection of single hits from the dataset of 1.9 × 10^5^ diffraction patterns produced a new dataset containing 1393 diffraction patterns (Li *et al.*, 2020 ▸). From this selection, 574 diffraction patterns are also present in our EM-based selection. The PSD function for the manual selection shown in Fig. 3 ▸(*c*) has a lower contrast without visible fringes. We attribute this mostly to the absence of the size-filtering step in manual selection. The virus size fluctuations could be caused by a slight change in hydration-layer thickness, variation of the position where the particles interact with the X-ray beam, slightly upstream or downstream of the focus, or a real sample size distribution of the PR772 virus. We note that in our previous work (Rose *et al.*, 2018 ▸) the number of single-hit diffraction patterns was ∼7.3 × 10^3^, which is about one order of magnitude higher than in this work. The reason for the smaller number of patterns was a combination of downtime owing to detector troubleshooting and time needed for sample-injection optimizations. The GDVN flow and pressures needed to be optimized because of the relatively large initial droplet diameters of ∼1–2 µm.

## 5. Orientation determination and background subtraction   

**Figure 4:**
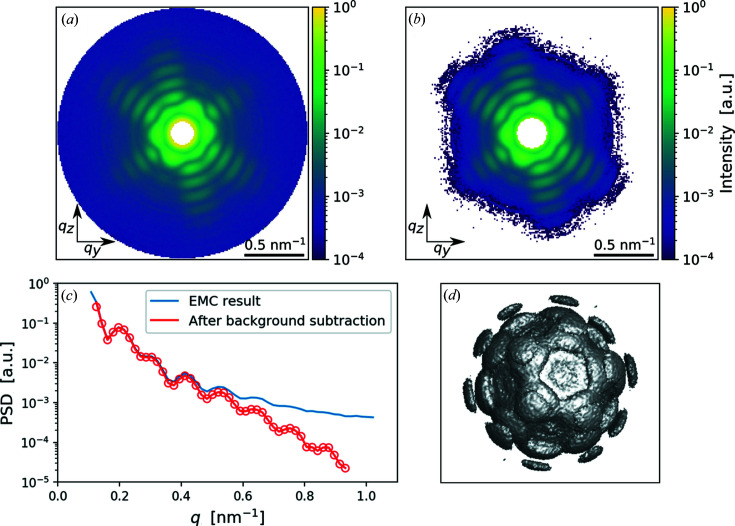
Orientation-determination results. (*a*) A 2D *q_y_–q_z_* cut of the 3D intensity distribution in reciprocal space and (*b*) the same intensity distribution after the background subtraction. (*c*) The PSD function for the EMC result (blue line) and after the background subtraction (red line with dots). It is well visible that the background subtraction enhanced structural visibility in the high *q* region. (*d*) The 3D intensity distribution in reciprocal space after the background subtraction, shown at 0.5% level of the maximum value.

For orientation determination we used the EMC algorithm implemented in the *Dragonfly* software package (Ayyer *et al.*, 2016 ▸). This iterative algorithm successfully combines 2D diffraction patterns into the 3D intensity distribution of the PR772 virus shown in Fig. 4 ▸(*a*) (see the Supporting information for further details).

The results presented in Fig. 4 ▸(*a*) clearly show that the recovered 3D diffraction intensity contains a substantial high-momentum background that may be caused by scattering on helium from the carrier gas. To reduce the influence of this background contribution, a background subtraction was applied. Several approaches for the background correction in SPI experiment analysis were developed earlier (Rose *et al.*, 2018 ▸; Lundholm *et al.*, 2018 ▸; Ayyer *et al.*, 2019 ▸) and it was understood that the background-correction method may affect the reconstructed structure. Here we used a combined approach for the background determination, first a constant background was subtracted and then, on the reconstruction step, the contrast of diffraction patterns was additionally enhanced by applying deconvolution algorithms (see the next section[Sec sec6] for details). In the first step, the background level was defined as the mean signal value in the high-momentum region of the 3D intensity distribution, free of particle-scattering contributions (see the Supporting information for details). Reciprocal-space data with and without background subtraction are shown in Figs. 4 ▸(*a*)–4 ▸(*c*). As seen in Fig. 4 ▸(*c*), the power-law dependence of data after the background subtraction is the same up to a high *q* range of about one inverse nanometre. This is in contrast to the data after EMC orientation determination [the blue curve in Fig. 4 ▸(*c*)], which are clearly saturated at high *q* range.

As a result of the background subtraction, more features and higher contrast were revealed in the high momentum-transfer region. The fringe visibility or averaged contrast 〈γ〉 was defined as
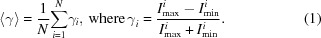
Here γ_*i*_ is the local contrast, 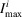 and 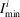 are the PSD-function values in the local maxima and following minima, respectively, and *N* is a number of pairs of maxima and minima considered in this analysis. In our case, the average contrast values 〈γ〉 were calculated for the first *N* = 6 pairs. For the experimental data before the background subtraction, we obtained the value 〈γ〉 = 0.41. The fringe contrast after the background subtraction showed significant improvement with 〈γ〉 = 0.58.

To proceed further with the analysis, low (*q* < 0.12 nm^−1^) and high (*q* > 0.93 nm^−1^) momentum-transfer regions were eliminated from the final data selection owing to scattering artefacts present in these regions. After the background subtraction, negative values of intensity were set to zero. A 3D intensity distribution in reciprocal space after the background subtraction is shown in Fig. 4 ▸(*d*).

## 6. Phase retrieval and PR772 virus structure   

To determine the electron-density distribution of the PR772 virus, the phase retrieval was performed using the 3D intensity distribution of the virus in reciprocal space described in the previous section[Sec sec5] [see Fig. 4 ▸(*d*)]. Iterative phase-retrieval algorithms are based on the Fourier transform between real and reciprocal/diffraction space using two constraints: in reciprocal space the amplitude of the signal is set equal to the experimentally measured values and in real space a finite support of the particle is used (Fienup, 1982 ▸; Marchesini, 2007 ▸).

The electron-density distribution of the PR772 virus was obtained with the following steps. First, the central gap, originating from the initial data masking of the diffraction patterns, was filled [see Figs. 4 ▸(*a*) and 4 ▸(*b*)]. This was accomplished by running multiple 3D reconstructions of the virus and leaving the central gap of the diffraction volume to freely evolve. All reconstructions produced practically identical continuous 3D functions in reciprocal space that smoothly covered the central gap (see Fig. S10). This reciprocal-space distribution with the filled central part was used for the next reconstruction step. On that step, 50 successive reconstructions using a combination of continuous hybrid input–output (Fienup, 2013 ▸), error reduction (Fienup, 1982 ▸) and shrink-wrap algorithms (Marchesini *et al.*, 2003 ▸) were performed. In addition to these algorithms, we used the Richardson–Lucy deconvolution technique (Clark *et al.*, 2012 ▸). Application of this method allowed us to take into consideration additional background and substantially enhance the contrast of the reconstructed diffraction pattern to the value of 〈γ〉 = 0.87. As a result, we obtained complex-valued real-space images for each of the 50 reconstructions (see the Supporting information for details).

**Figure 5:**
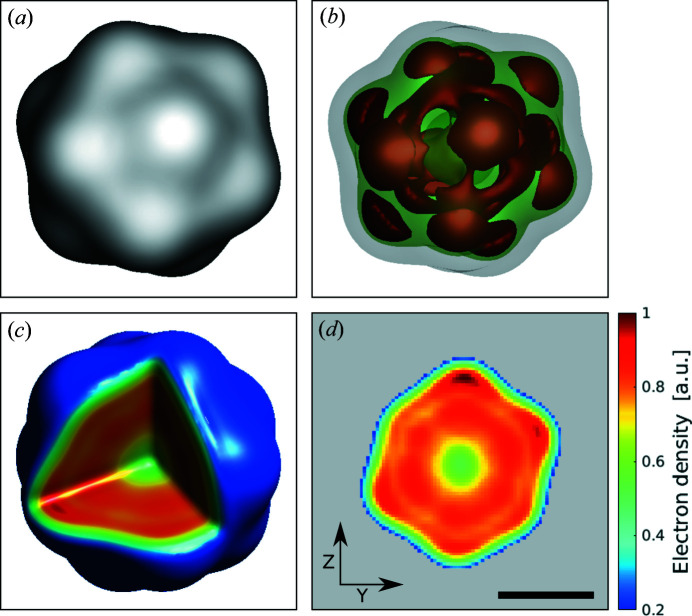
Electron density of the reconstructed PR772 virus normalized to the maximum value. (*a*) The outer structure of the PR772 virus at the isosurface value of 20% of the maximum electron density. (*b*) The inner 3D structure of the PR772 virus at the isosurface values of 85% (brown area), 75% (green area) and 20% (gray area). (*c*) A 3D section of the virus. (*d*) An electron-density slice of the virus. Amplitude values of less than 0.2 were set to gray color scale. The color map for (*c*) and (*d*) is the same. Images were up-sampled three times for better visibility. The scale bar in (*d*) is 30 nm.

On the final step, to identify the electron density of the virus we used mode decomposition. We first introduced a density matrix in the form
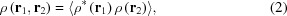
where ρ(**r**) relates to complex-valued real-space images and the brackets 〈…〉 indicate ensemble averaging over different reconstructions. We further decomposed the density matrix into orthogonal modes ρ_*n*_(**r**) using principal component analysis (Khubbutdinov *et al.*, 2019 ▸):
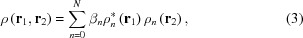
where β_*n*_ refers to eigenvalues of this decomposition. This approach is advantageous in comparison with averaging that would make important object features blurry and possibly affect the final resolution. By considering the zero mode only, unique features present in all other reconstructions will be represented. In practice, we used all 50 reconstruction results and performed mode decomposition. The fundamental mode (with the weight factor β_0_ of 99%) was considered as the final result of reconstruction. Such a high weight-factor value indicates that all reconstructions converged essentially to the same result with an uncertainty level of only 1%. To determine the electron density, we took an absolute value of this fundamental mode complex-valued image. A three-times up-sampled version of the real-space electron density is shown in Fig. 5 ▸ (see the Supporting information for details).

As seen in Figs. 5 ▸(*a*)–5(*c*), the retrieved electron density of bacteriophage PR772 shows the expected icosahedral structure. A 2D cut of the virus structure is presented in Fig. 5 ▸(*d*), where a higher electron density in a thin outer shell is well resolved and is attributed to the capsid proteins arrangement. As can be seen in Fig. 5 ▸(*d*), the electron density in the center of the reconstructed virus particle is reduced. The reason for this may be the heterogeneity of virus particles present in solution and injected in the X-ray beam.

Bacteriophage PR772, like other members of the *Tectiviridae* family, contains an inner proteolipid membrane that facilitates delivery of the viral genomic DNA during infection (Mäntynen *et al.*, 2019 ▸). When the virus binds to a bacterial host cell the inner membrane is extruded from one of the viral vertices to form a nanotube that facilitates genome delivery. Spontaneous release of the viral genome has been reported (Peralta *et al.*, 2013 ▸; Santos-Pérez *et al.*, 2017 ▸). Our bacteriophage PR772 preparations were also analyzed by cryo-EM (Fig. S6). Under the conditions used for plunge freezing, we observed particles that were more rounded than icosahedral. Preliminary volume analysis suggests that some particles had released their DNA. These particles appeared to be ‘triggered’ during the freezing process since transmission electron microscopy imaging did not reveal this. It may be possible from minor differences in virus preparation or upon XFEL sample delivery that some of the virus particles were similarly ‘triggered’ to release their genomes during cryo-freezing, thus resulting in a decrease in inner electron density. Previous analyses of XFEL snapshots on the same virus also suggested that some particles exhibit decreased inner density (Shi *et al.*, 2019 ▸; Hosseinizadeh *et al.*, 2017 ▸).

**Figure 6:**
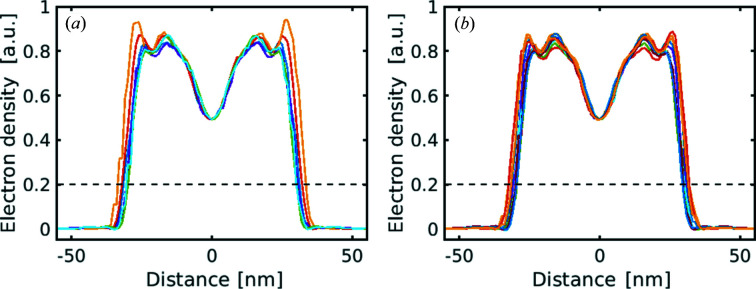
Electron-density profiles of the reconstructed virus PR772 normalized to the maximum value for the cut between vertices (*a*) and facets (*b*). The horizontal black dashed lines denote a particle-size threshold of 0.2. The mean virus size is 63 and 61 nm for the distance between vertices and between facets, respectively.

To identify the particle size, the electron-density profiles in different directions were investigated. In this work, we used the same approach as developed earlier (Rose *et al.*, 2018 ▸) and analyzed the electron-density profiles in the directions from facet to facet and from vertex to vertex of the reconstructed virus particle [see Figs. 6 ▸(*a*) and 6 ▸(*b*)]. For the particle-size estimate, we selected the electron-density threshold value of 0.2 as it was considered in the shrink-wrap algorithm during the phase retrieval. From this criterion we determined the particle size in different directions (see Table 2 ▸ and the Supporting information). Thus, the obtained mean particle size was 61 ± 2 nm between facets and 63 ± 2 nm between vertices. These sizes correspond well to the initial range of particle sizes (from 55 to 84 nm) considered at the initial classification step (see Section 4) and other XFEL data performed on the same virus (Kurta *et al.*, 2017 ▸; Rose *et al.*, 2018 ▸).

Similar to previous work (Kurta *et al.*, 2017 ▸; Rose *et al.*, 2018 ▸), we also observed a certain elongation of the particle shape, which might be inherently present in the viruses in solution or appear owing to the aerosol injection conditions. We defined the elongation of the particle by the following measure,
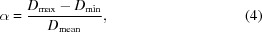
where *D*_max_, *D*_min_ and *D*_mean_ are maximum, minimum and mean particle sizes, respectively. The virus structure obtained in the current work showed an elongation value of α = 11% for sizes taken between vertices, which is similar to the result of the previous SPI experiments (Rose *et al.*, 2018 ▸) with α = 9%.

The mean capsid thickness was obtained from the Gaussian fitting of the electron-density profiles with well pronounced features (Fig. S14) and was determined to be 7.6 ± 0.3 nm. The thickness of the capsid in recent cryo-EM studies (Reddy *et al.*, 2019 ▸) was identified to be below 10 nm, which is in good agreement with the thickness determined in this experiment.

**Figure 7:**
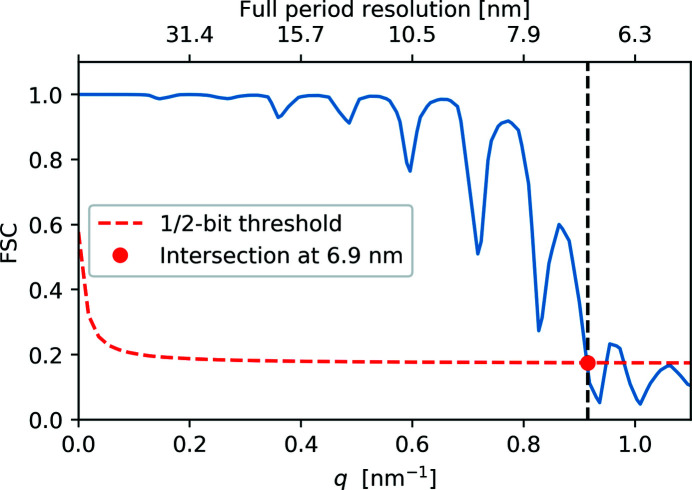
FSC of the final reconstruction (blue line) that shows a 6.9 nm resolution (red dot) with a half-bit threshold (red dashed line).

Finally, we determined the resolution of the reconstructed electron density of the PR772 virus. We used the Fourier shell correlation (FSC) approach (Harauz & van Heel, 1986 ▸) to determine the resolution of the reconstructed virus. For this method two independent sets of reconstruction were required, usually each is based on half of the available dataset. FSC measures the normalized cross-correlation coefficient between both reconstructions over corresponding shells in Fourier space. The results of the FSC analysis are shown in Fig. 7 ▸. To estimate the achieved resolution, we used half-bit threshold criteria (van Heel & Schatz, 2005 ▸). Its intersection with the FSC curve gave a resolution value of 6.9 nm (see Fig. 7 ▸). The obtained result is better than the previously reported value of ∼8 nm for the same virus (Rose *et al.*, 2018 ▸), though the number of diffraction patterns used for the final analysis was much lower (15% of the previous dataset). In this case, the resolution was limited by the number of diffraction patterns and moderate scattered intensity, whereas previous reconstructions were limited by the extent of the detector.

## 7. Summary and outlook   

We have presented an implementation of the SPI data-analysis workflow from diffraction patterns measured at the AMO instrument at the LCLS to reconstruct the electron density of bacteriophage PR772. This work is an important methodological step for development of SPI data analysis. We implemented several methods into the workflow including EM-based classification and mode decomposition that were crucial for the high-resolution final reconstruction. Although only half of the detector was operational, implementation of all of these steps allowed us to determine the PR772 virus structure with a higher resolution as compared with previous SPI studies.

From the initial set of 1.2 × 10^7^ experimentally measured diffraction patterns, the final single-hit selection set contained 1085 diffraction patterns. About 53% of this final set was also present in the single-hit selection made manually. The number of diffraction patterns classified for further analysis is substantially lower than in the previous experiment (Rose *et al.*, 2018 ▸), improvements in sample delivery to increase the number of diffraction patterns are, therefore, crucial for advancements in reconstruction resolution.

The combination of all methods implemented in the workflow allowed us to obtain the 3D electron density of the virus with a resolution of 6.9 nm based on the FSC analysis, which is better than that obtained in the previous studies (Rose *et al.*, 2018 ▸). The obtained mean PR772 virus size in this experiment was 61 nm (between facets) and 63 nm (between vertices), like in the previous SPI experiments performed on the same PR772 virus (Kurta *et al.*, 2017 ▸; Rose *et al.*, 2018 ▸). We also observed a similar elongation of ∼11% of the virus structure as was determined in the previous experiments.

The presented research is another step forward in SPI data analysis. Implemented steps may become especially important when SPI experiments are performed at high repetition rate XFELs, such as the European XFEL (Decking *et al.*, 2020 ▸; Mancuso *et al.*, 2019 ▸) and LCLS-II (Schoenlein *et al.*, 2015 ▸) facilities. The first experiments performed at the European XFEL demonstrated the possibility of collecting the SPI data at a megahertz rate (Sobolev *et al.*, 2020 ▸), which might be crucial for future progress in SPI experiments performed at XFELs.

## Supplementary Material

Supporting information. DOI: 10.1107/S2052252520012798/it5022sup1.pdf

Supporting Movie: a 3D intensity distribution of the PR772 virus. DOI: 10.1107/S2052252520012798/it5022sup2.avi

Supporting Movie: the shape structure of the PR772 virus. DOI: 10.1107/S2052252520012798/it5022sup3.avi

Supporting Movie: the 3D inner structure of the PR772 virus. DOI: 10.1107/S2052252520012798/it5022sup4.avi

Supporting Movie: a visualization of the 2D structure of the PR772 virus. DOI: 10.1107/S2052252520012798/it5022sup5.avi

## Figures and Tables

**Table 1:** Datasets selected at different stages of the analysis: hit finding selection, PSD-fitting quality filtering, particle-size filtering and single-hit diffraction pattern selection The percentage of the chosen dataset to the initial one S_0_ is given in parentheses.

Analysis step	Dataset name	Number of diffraction patterns
Initial dataset	S_0_	1.2 × 10^7^
Hit-finding classification	S_hit_	191183 (1.6%)
PSD-fitting score filtering	S_fit_	179886 (1.5%)
Particle-size filtering	S_D_	18213 (0.1%)
First EM-based classification	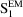	1609
Second EM-based classification	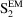	1402
Third EM-based classification	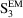	1366
Fourth EM-based classification	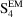	1401
Fifth EM-based classification	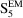	2119
Final EM-based classification	S_EM_	1085 (0.009%)
Manual selection	S_man_	1393 (0.01%)

**Table 2:** Particle-size analysis from facet to facet and from vertex to vertex for the reconstructed PR772 virus All distances between facets and vertices are given in the Supporting information.

		Size (nm)
Facet to facet	*D*_mean_	61 ± 2
	*D*_max_	64 ± 2
	*D*_min_	59 ± 2
Vertex to vertex	*D*_mean_	63 ± 2
	*D*_max_	67 ± 2
	*D*_min_	60 ± 2
