# An advanced workflow for single-particle imaging with the limited data at an X-ray free-electron laser. Corrigendum

**DOI:** 10.1107/S2052252522000501

**Published:** 2022-03-01

**Authors:** Dameli Assalauova, Young Yong Kim, Sergey Bobkov, Ruslan Khubbutdinov, Max Rose, Roberto Alvarez, Jakob Andreasson, Eugeniu Balaur, Alice Contreras, Hasan DeMirci, Luca Gelisio, Janos Hajdu, Mark S. Hunter, Ruslan P. Kurta, Haoyuan Li, Matthew McFadden, Reza Nazari, Peter Schwander, Anton Teslyuk, Peter Walter, P. Lourdu Xavier, Chun Hong Yoon, Sahba Zaare, Viacheslav A. Ilyin, Richard A. Kirian, Brenda G. Hogue, Andrew Aquila, Ivan A. Vartanyants

**Affiliations:** a Deutsches Elektronen-Synchrotron DESY, Notkestraße 85, Hamburg, D-22607, Germany; b National Research Center ‘Kurchatov Institute’, Akademika Kurchatova pl. 1, Moscow, 123182 Russian Federation; c National Research Nuclear University MEPhI (Moscow Engineering Physics Institute), Kashirskoe sh. 31, Moscow, 115409, Russian Federation; d Department of Physics, Arizona State University, Tempe, Arizona AZ 85287, USA; e School of Mathematics and Statistical Sciences, Arizona State University, Tempe, Arizona AZ 85287, USA; f Institute of Physics, ELI Beamlines, Academy of Sciences of the Czech Republic, Prague, CZ-18221, Czech Republic; g Australian Research Council Centre of Excellence in Advanced Molecular Imaging, Department of Chemistry and Physics, La Trobe Institute for Molecular Science (LIMS), La Trobe University, Melbourne, Victoria 3086, Australia; h School of Life Sciences, Arizona State University, Tempe, Arizona AZ 85287, USA; i Biodesign Institute Center for Immunotherapy, Vaccines and Virotherapy, Arizona State University, Tempe, Arizona AZ 85287, USA; j Stanford PULSE Institute, SLAC National Accelerator Laboratory, 2575 Sand Hill Road, Menlo Park, CA 94025, USA; k Department of Molecular Biology and Genetics, Koc University, Istanbul, 34450, Turkey; l Center for Free Electron Laser Science (CFEL), DESY, Notkestraße 85, Hamburg, D-22607, Germany; m Laboratory of Molecular Biophysics, Department of Cell and Molecular Biology, Uppsala University, Husargatan 3, Uppsala, SE-75124, Sweden; n SLAC National Accelerator Laboratory, 2575 Sand Hill Road, Menlo Park, CA 94025, USA; o European XFEL, Holzkoppel 4, Schenefeld, D-22869, Germany; p Physics Department, Stanford University, 450 Jane Stanford Way, Stanford, CA 94305-2004, USA; q School for Engineering of Matter, Transport and Energy, Arizona State University, Tempe, AZ 85287, USA; r University of Wisconsin Milwaukee, Milwaukee WI 53211, USA; s Moscow Institute of Physics and Technology, Moscow, 141700, Russian Federation; t Max-Planck Institute for the Structure and Dynamics of Matter, Luruper Chaussee 149, Hamburg, D-22761, Germany; u Biodesign Institute, Center for Applied Structural Discovery, Arizona State University, Tempe, AZ 85287, USA

**Keywords:** coherent X-ray diffractive imaging (CXDI), free-electron lasers, single particles, XFEL

## Abstract

An error in Fig. 3 ▸(*c*) of the article by Assalauova *et al.* [*IUCrJ* (2020), **7**, 1102–1113] is corrected.

## Abstract 2

An error in the article by Assalauova *et al.* [*IUCrJ* (2020), **7**, 1102–1113] is corrected.

In Fig. 3 ▸(*c*) of the article by Assalauova *et al.* (2020 ▸), the values of PSD function for the manual single-hit selection were not correct (see corrected figure below). This update does not affect any conclusions made in the original paper.

## Figures and Tables

**Figure 3:**
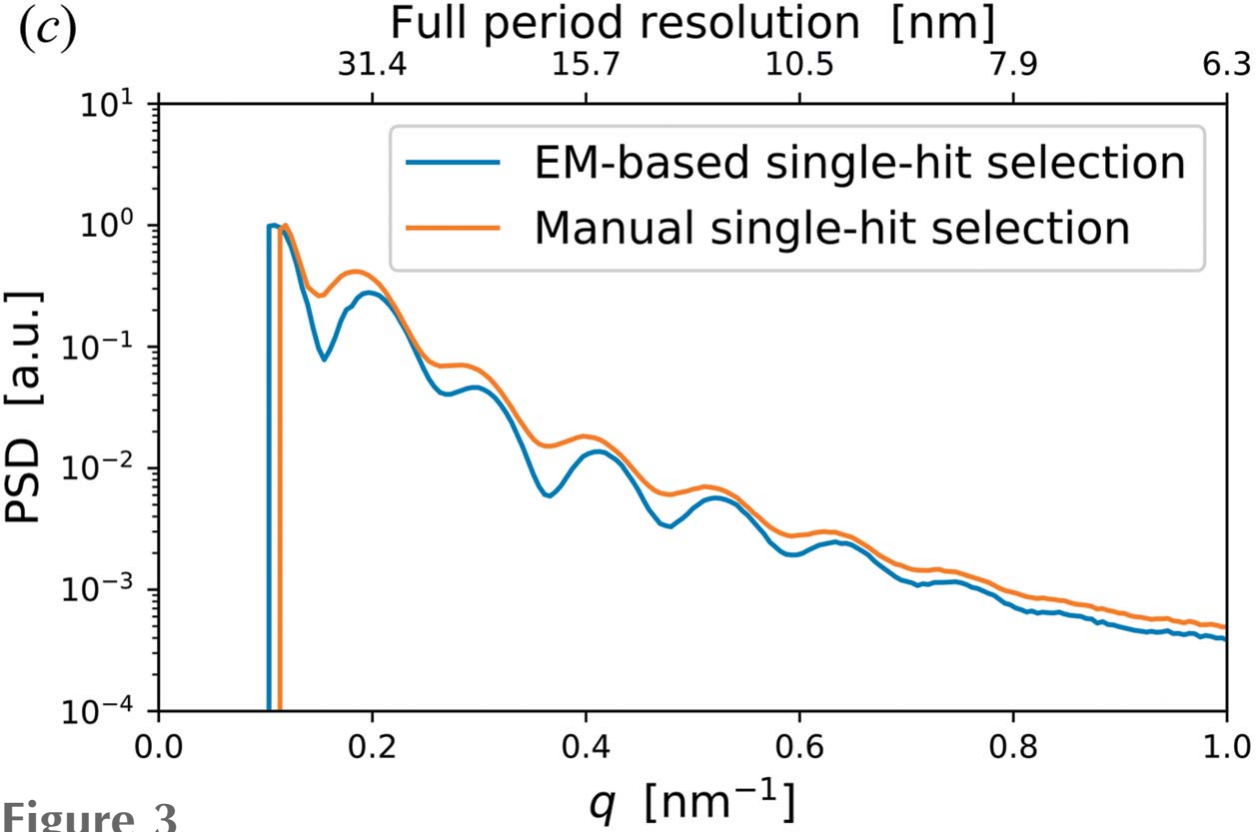
Classification of diffraction patterns by EM clustering. (*c*) Averaged PSD functions for EM-based single-hit selection containing 1085 patterns (blue line) and for manual selection containing 1393 patterns (orange line).
