# Supplementary material for: An advanced workflow for single-particle imaging with the limited data at an X-ray free-electron laser
Source: IUCrJ. 2020 Oct 15;7(Pt 6):1102–13. doi: 10.1107/S2052252520012798 (PMC7642788; doi:10.1107/S2052252520012798)
Supplement: Supplementary file 1 [file m-07-01102-sup1.pdf]

IUCrJ

Volume 7 (2020)

Supporting information for article:

An advanced workflow for single-particle imaging with the limited data at an X-ray free-electron laser

Dameli Assalauova, Young Yong Kim, Sergey Bobkov, Ruslan Khubbutdinov, Max Rose, Roberto Alvarez, Jakob Andreasson, Eugeniu Balaur, Alice Contreras, Hasan DeMirci, Luca Gelisio, Janos Hajdu, Mark S. Hunter, Ruslan P. Kurta, Haoyuan Li, Matthew McFadden, Reza Nazari, Peter Schwander, Anton Teslyuk, Peter Walter, P. Lourdu Xavier, Chun Hong Yoon, Sahba Zaare, Viacheslav A. Ilyin, Richard A. Kirian, Brenda G. Hogue, Andrew Aquila and Ivan A. Vartanyants

S1. Analysis of additional instrumental scattering

Visual inspection of the measured diffraction patterns showed an additional scattering signal close to the central part of the beam. This signal remains stable from pulse to pulse, which indicates that it most probably originates from beamline scattering. This additional instrumental scattering can be well seen on the averaged diffraction pattern in one of the experimental runs (see Fig. S1(a)).

We analyzed histograms of intensity for individual pixels and noticed that pixels with additional instrumental scattering most often recorded a signal of several photons. Contrary to that, pixels without this additional scattering most frequently recorded a signal of zero photons. We assumed that beamline scattering follows a Gaussian distribution and it was incoherently added to particle scattering. To correct this additional signal, we fit the first peak on histogram of intensity for each pixel by a Gaussian function (see Fig. S2). Then we subtract the value of the Gaussian center from the total signal of this pixel for all diffraction patterns. This instrumental scattering subtraction was crucial for further beam center position finding and particle size filtering. We did not mask this region because we would lose important information about the first diffraction minimum.

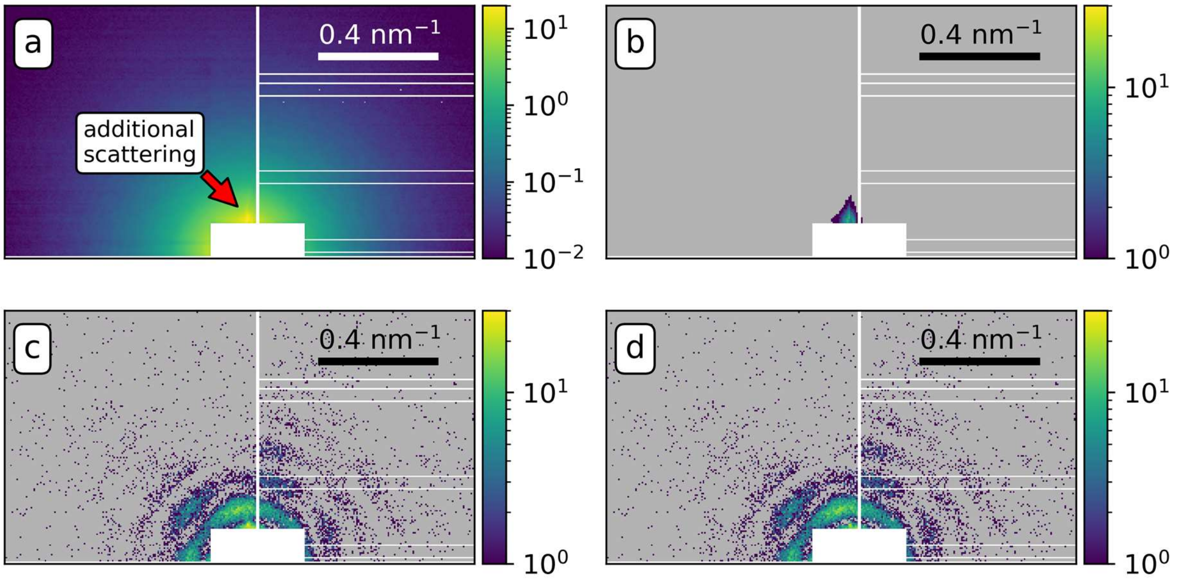

Figure S1 (a) An averaged diffraction pattern of one of the runs. White regions in the diffraction patterns correspond to a mask introduced to hide misbehaving pixels. Additional instrumental scattering originating from the beamline is well visible in the central part of the averaged diffraction pattern. (b) Identified additional scattering for this run. Diffraction pattern before (c) and after (d) subtraction of additional scattering.

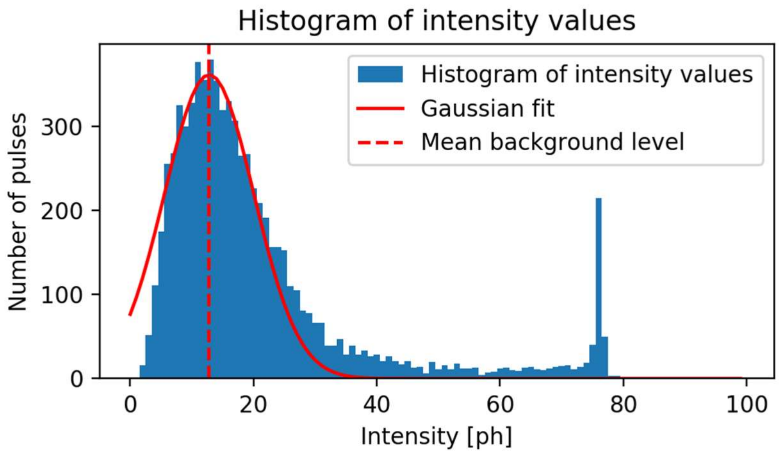

Figure S2 Histogram of intensity values for a selected pixel from one of the runs with strong additional instrumental scattering. The pixel shows the most frequently recorded value is 13 photons. This histogram was fitted with a Gaussian function and the mean value of this Gaussian function was subtracted from all intensity values for different pulses corresponding to that pixel. The peak on the right side of the histogram is due to limitations of the detector: if the detector pixel collects more than 75 photons, its response is always in the range of 76-79 photons.

S2. Beam center position finding

In the present work, the beam center position was retrieved from the diffraction patterns. Detector consists of two identical panels with the gap between them for direct beam propagation. Due to the fact that the signal from only one panel was available, the beam center position could not be determined by centrosymmetric property of diffraction patterns. Furthermore, the detector panel was moved during the experiment, and we estimated the beam position twice – before and after the detector panel was moved. The beam center position was determined in the following way. First, the sum of all diffraction patterns was calculated. The resulting average diffraction pattern was rotationally symmetric and allowed a rough estimate of the beam position center. For the success of this step, it was crucial to subtract parasitic scattering from the beamline as described in the previous section. To define the beam position center more carefully on the next step, diffraction patterns with a narrow distribution of particle sizes were selected and the averaged diffraction pattern was obtained. This diffraction pattern has pronounced diffraction fringes and it was correlated with the two-dimensional (2D) form factor of a spherical particle (see Fig. S3). Inspection of this method on simulated data with similar parameters showed that mean deviation of the refined center from the true center of diffraction patterns is less than half of a pixel.

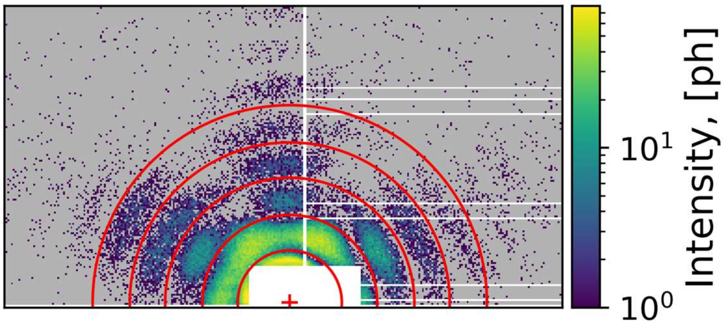

Figure S3 Center position on a selected diffraction pattern. Minima of the optimal spherical form factor are shown by red circles.

S3. Particle size filtering

The particle size filtering was based on the fitting of the power spectral density (PSD) function of each diffraction pattern with the form factor of a sphere. A set of form factors corresponding to the spheres with the diameter in the range from 30 to 300 nm was generated first. On the next step the PSD function of each diffraction pattern was fitted with a spherical form factor function from the generated set (see Fig. S4(a)). As the fit quality measure for the certain size (diameter) of the spherical particle, the mean difference was used

$$D_S = \frac{1}{q_{max}-q_{min}} \sum_{q_{min}}^{q_{max}} \left| I_{exp}(q) - I_s(q) \right|, \text{ (S1)}$$

where $I_{exp}(q)$ is the PSD value of the experimental intensity for selected q, $I_s(q)$ is the form factor of a sphere with the size (diameter) S. In equation (S1) the q-values were ranging from q_{min}=0.12/0.15 nm^{-1} before and after the detector panel was moved, up to q_{max}=0.66 nm^{-1}. An example of the mean difference function of equation (S1) obtained for one of the diffraction patterns is shown in the Fig. S4(b). This function has several minima, where the first minimum corresponds to a sphere with the best size. The second minimum corresponds to a sphere with the second-best size, *etc.* To measure fidelity of the particle size estimation we used fidelity score (*FS*) defined as

$$FS = \frac{D_{S_2}}{D_{S_1}}, \text{ (S2)}$$

where D_{S_1} and D_{S_2} are the values of the mean difference function D_S corresponding to the first and second minima in Fig. S4(b). The fidelity score histogram for all diffraction patterns identified as hits (1.9×10^5 diffraction patterns) is shown in Fig. S5. According to its definition in Eq. (S2), if the fidelity score is equal to unity (FS = 1) it means that D_S values equal for two different minima, therefore fitting cannot find an appropriate size for a particle that will correspond to a given diffraction pattern. We introduced a threshold value of FS = 1.05 and considered all diffraction patterns with the fidelity score

higher than this value (see Fig. S5). By that we determined 1.8×10^5 diffraction patterns that were selected for the further particle size filtering described in the main text.

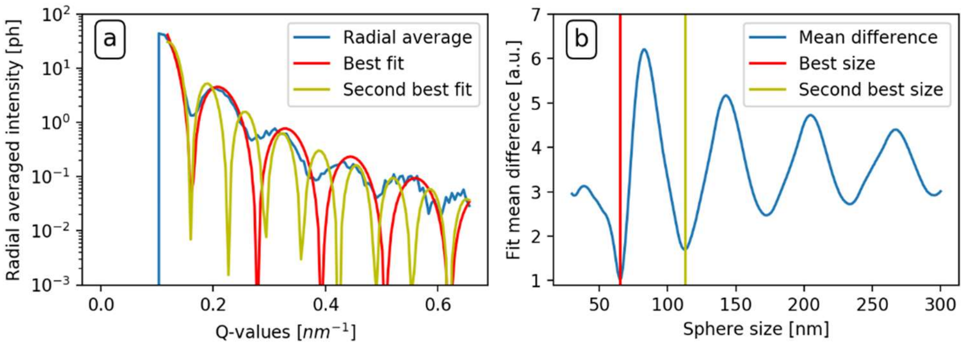

Figure S4 PSD fitting analysis of the diffraction pattern. (a) PSD function (blue line) was fitted with the form factors of spherical particles of different size. Red and yellow lines correspond to the form factors of the spherical particles with the best and second best size, that were used for calculation of fidelity score. (b) Mean difference function as defined in Equation (S1). Fidelity score value is 1.6 for this diffraction pattern.

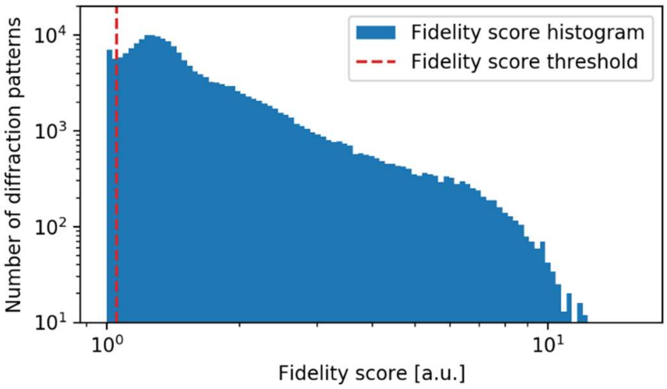

Figure S5 Fidelity score histogram for all diffraction patterns identified as hits in the experiment. Fidelity score threshold of the value 1.05 is shown as the vertical dashed red line. 1.8×10^5 selected diffraction patterns with fidelity score above threshold were used for further analysis.

Virus size distribution according to the cryo-EM studies of the PR772 virus used in the experiment is shown in Fig. S6.

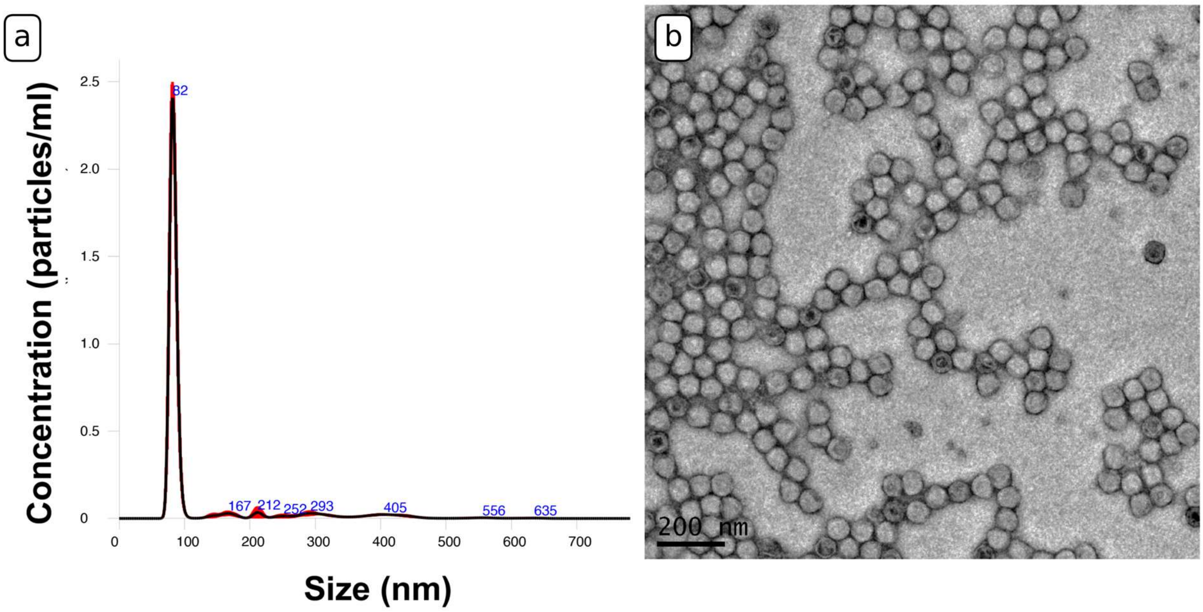

Figure S6 Cryo-EM structural studies of PR772 used in the experiment. (a) Virus size distribution profile. (b) PR772 visualization with screening transmission electron microscopy (TEM).

After the size filtering and running EM algorithm, we ended with the data set containing 1,085 patterns (see Table 1 of the main text). In order to identify performance of single particle collection as well as efficiency of the 3D printed nozzles we plot a histogram of selected patterns as a function of experimental run (see Fig. S7). This histogram shows that collection was significantly improved towards the end of the experiment.

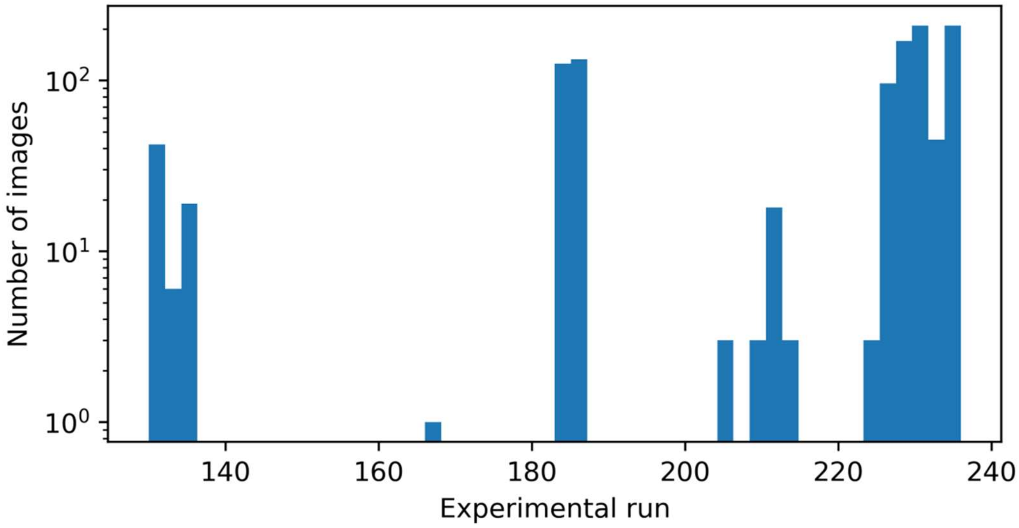

Figure S7 Histogram of the number of images per experimental run from the data set containing 1,085 patterns.

S4. Orientation determination and background subtraction

Before orientation determination we defined the region of interest for the collected data. This was performed twice, before and after the detector panel was moved. For the first/second detector position the detector area from $q_{\min}$=0.18/0.23 nm^{-1} to $q_{\max}$=1.03 nm^{-1} was considered. The data at $q < q_{min}$ was excluded from orientation determination due to poor convergence, but these data were used to compute the 3D amplitude in reciprocal space. The data corresponding to $q > q_{\max}$ were removed from the analysis because the scattering signal is indistinguishable from noise. The selected data was binned 2 by 2 due to computing memory constraints. The diffraction patterns were then converted into Dragonfly (Ayyer *et al.*, 2016) input format using exact coordinates of each pixel, two retrieved beam center positions (before and after detector panel was moved), and other experimental parameters *such as* the wavelength of 7.3 Å and the distance from the interaction region to the detector of 130 mm. Orientation determination was performed with quaternion sampling of 10, the starting beta value 0.01 which was multiplied by 1.3 every 30 iterations. We performed 300 iterations with the EMC algorithm and took iteration 270 as the best one (Fig. S8).

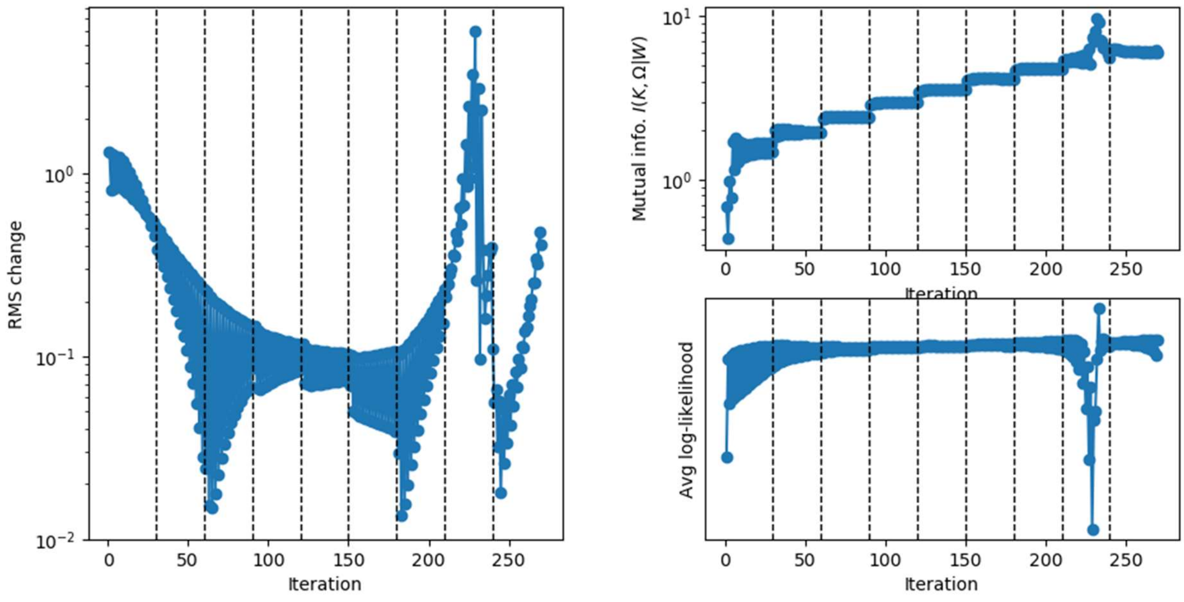

Figure S8 Screenshot of 'Dragonfly' software showing diagnostics of each 3D orientation reconstruction. (a) R.M.S. change indicates the degree of model modification at each iteration. (b) Mutual information between model tomograms and experimental data as the metric of the 'sharpness' of the probability distribution over orientations. (c) Average log-likelihood of patterns given a model as a metric of how an iterative reconstruction approaches the global likelihood maximum.

Results of the EMC algorithm for orientation determination are shown in Fig. S8(a-c). It is well seen that at high q-signal level some background is still present after orientation determination.

To determine the background level, the signal in selected areas of the high q-region, where the contribution of the meaningful data is minimal, was analyzed (see Fig. S9(a-c)). The histogram of intensity in this area is shown in Fig. S9(d). The background level was defined as the mean signal value and was subtracted from the 3D intensity map in reciprocal space. Negative values of intensity in the final representation were set to zero.

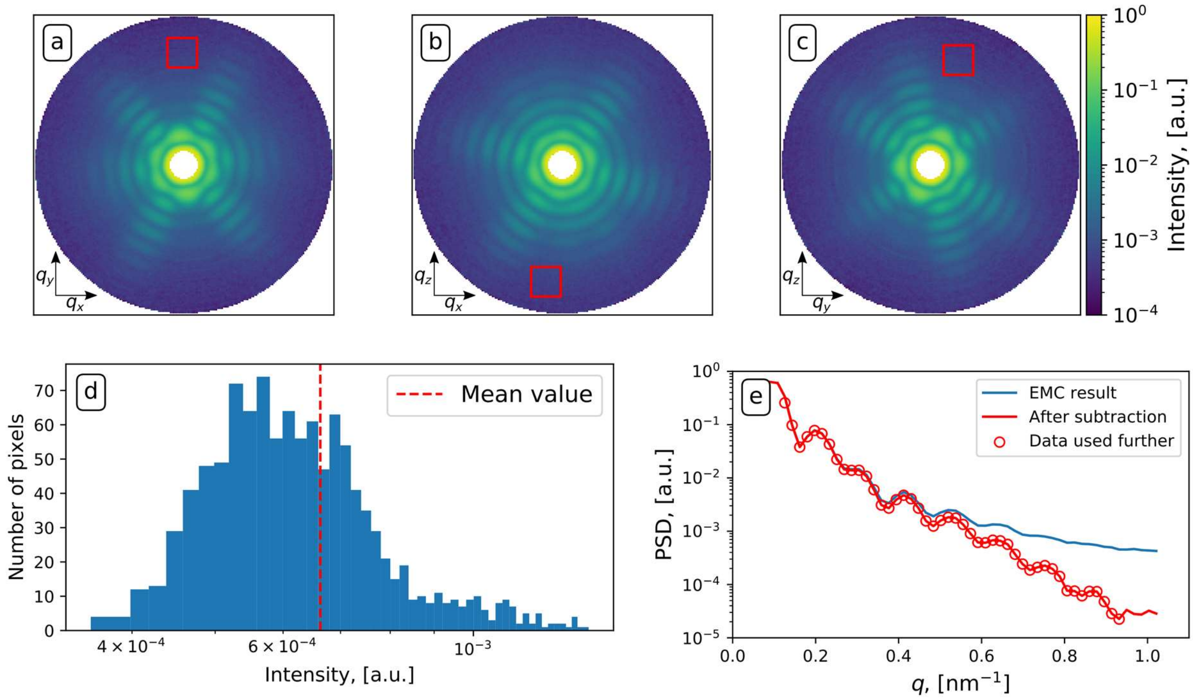

Figure S9 Results of the EMC orientation determination algorithm. (a-c) Orthogonal two-dimensional cuts through the center of the 3D volume of reciprocal space after application of the EMC algorithm. For the background estimate the intensity values in the region of high q shown with red squares were analyzed. (d) Histogram of the signal from the area shown in (a-c). The mean value of the signal is shown with the vertical dashed red line. (e) PSD functions before (blue line) and after (red line) background subtraction. To avoid artifacts at low and high q-values a part of the curve indicated with red dots was considered for further analysis.

The PSD function after background subtraction (red line in Fig. S9(e)) reveals artifacts in the regions of low ($q < 0.12$ nm^{-1}) and high ($q > 0.93$ nm^{-1}) momentum transfer values. Since the data in these regions did not follow the expected spherical form factor behavior, we did not consider this part of the data. Data used for further analysis are shown with red dots in Fig. S9(e). For visual inspection we show the final 3D intensity distribution of the PR772 virus in animation (see Supplementary Movie it5022sup1.avi).

S5. Phase retrieval

Phase retrieval and electron density determination of the PR772 virus was performed in several steps. First, due to masking, the central part of the 3D intensity map was missing (see Fig. S9(a-c)). To recover it, several reconstructions, with an assumption of free-evolving intensity in this part of reciprocal space, were performed. The following algorithms were considered at this stage: 90 iterations of cHIO with the feedback value 0.8 (Fienup, 2013), 200 iterations of the ER algorithm (Fienup, 1982) with alternation of the shrink-wrap algorithm each 10 iterations with the threshold value of 0.2 and Gaussian filtering with 3 to 2 sigma. (Marchesini *et al.*, 2003). This combination of algorithms was repeated three times for one reconstruction with the total number of 870 iterations. All obtained reconstructions showed identical central part and we used one of them in further analysis. In Fig. S10(a) the PSD functions of the initial and one of the reconstructed data are shown. One can see from that figure that the reconstructed curve follows very well the experimental data points. For the low q-values below 0.14 nm^{-1} the experimental data were substituted with the data obtained in phase retrieval. Difference between experimental data and reconstruction in the central fringe is contributed to incorrectly reduced detector signal for intensity above 75 photons (Fig. S2). This modified 3D intensity map was used for the final phase retrieval and virus structure determination (Fig. S10(b)).

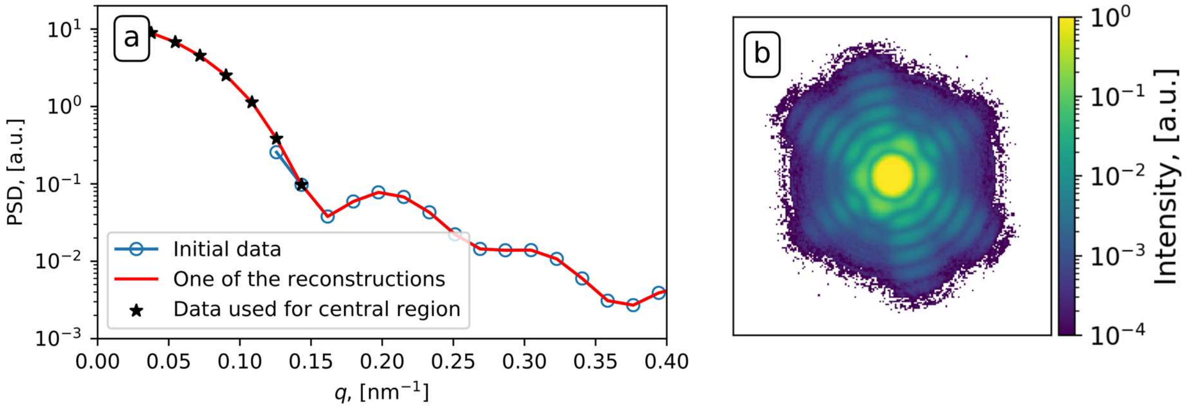

Figure S10 (a) PSD functions for experimental data (blue empty dots) and one of the reconstructions (red line). The central part below q = 0.14 nm^{-1} (black stars) was taken from this reconstruction for further analysis (b) Modified data with the filled central part. White area around the diffraction pattern is the part of reciprocal space where the data were set to zero initially but were allowed to freely evolve during the iterative phase retrieval.

On the next step 50 individual reconstructions were performed. In these reconstructions intensities at high q-values (in the regions where they were initially set to zero (white area in Fig. S10(b)) were allowed to freely evolve with a weight factor of 0.9. The initial support was taken as a Fourier transform of the 3D data used for reconstructions and had spherical shape with diameter about 90 nm.

The same sequence of algorithms was used for these reconstructions as mentioned above plus it was performed 100 iterations of the Richardson-Lucy algorithm (Clark *et al.*, 2012) with the total number of 970 iterations. This algorithm based on deconvolution technique allowed to additionally enhance the contrast of the reconstructed diffraction patterns to the value of $\langle\gamma\rangle$=0.87, and by that remove the remaining background from the 3D diffraction patterns, which is defined as point spread function (PSF) shown in Fig. S11. As a result, we obtained complex valued real space images for each 50 reconstructions from which the absolute value was considered as an electron density of the virus.

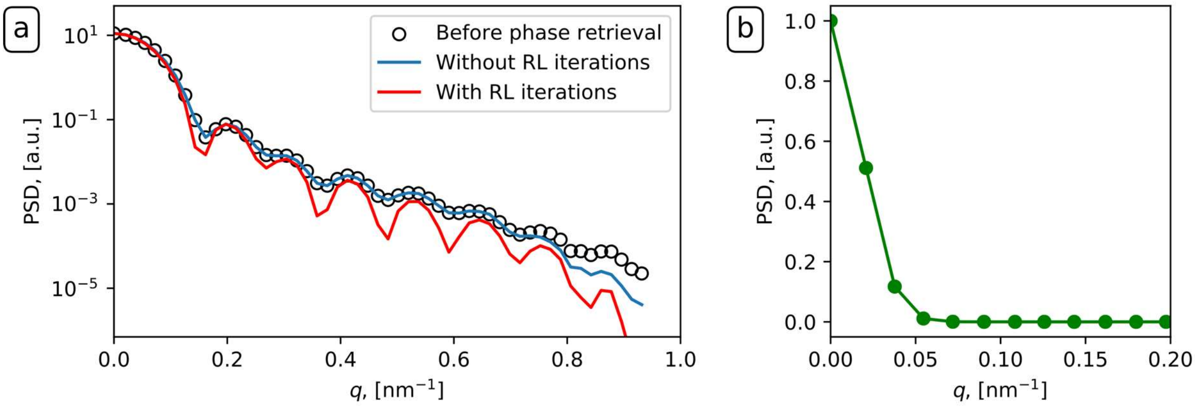

Figure S11 (a) PSD functions for data before phase retrieval (black empty dots) and one of the reconstructions without Richardson-Lucy iterations (blue line) and with them (red line). This additional deconvolution allowed us to improve contrast of diffraction patterns to the value of $\langle\gamma\rangle$=0.87. (b) PSF for individual reconstruction as a result of Richardson-Lucy deconvolution algorithm. Intensity is normalized to the maximum value.

S6. Mode decomposition and virus electron density analysis

To determine the final electron density of the virus, the mode decomposition of the reconstruction set was performed and as an outcome of this procedure an orthogonal set of modes was found. The whole procedure consists of the following steps (see Fig. S12 and (Khubbutdinov *et al.*, 2019)):

a) Initial 4D matrix (Fig. S12(a)) consists of 3D amplitudes of the reconstructions (203×203×203 pixels), where the fourth dimension is given by the number of reconstructions (50 in the present case).

b) This 4D matrix of amplitudes is rearranged into a 2D matrix (Fig. S12(b)) with 50 columns, where each 3D amplitude matrix was rearranged to a 1D column.

c) Next, the mode decomposition is performed for the density matrix that is obtained by multiplication of the previously defined 2D matrix transposed complex conjugated and 2D matrix itself (Fig. S12(c)). By diagonalization of this matrix using Principal Component Analysis (PCA), eigenfunctions and eigenvalues of the reconstructed object are obtained.

We considered the fundamental mode of the reconstruction set (with a weight of 99%) as a final result.

The final electron density of the PR772 virus was three times up-sampled for better visual impression. The comparison between the initial and up-sampled structures is shown in Fig. S13. The electron density of the reconstructed PR772 virus was normalized to the maximum value in this figure. For visual inspection we also show the final virus structure (outer, inner and through x-axis) in animations.

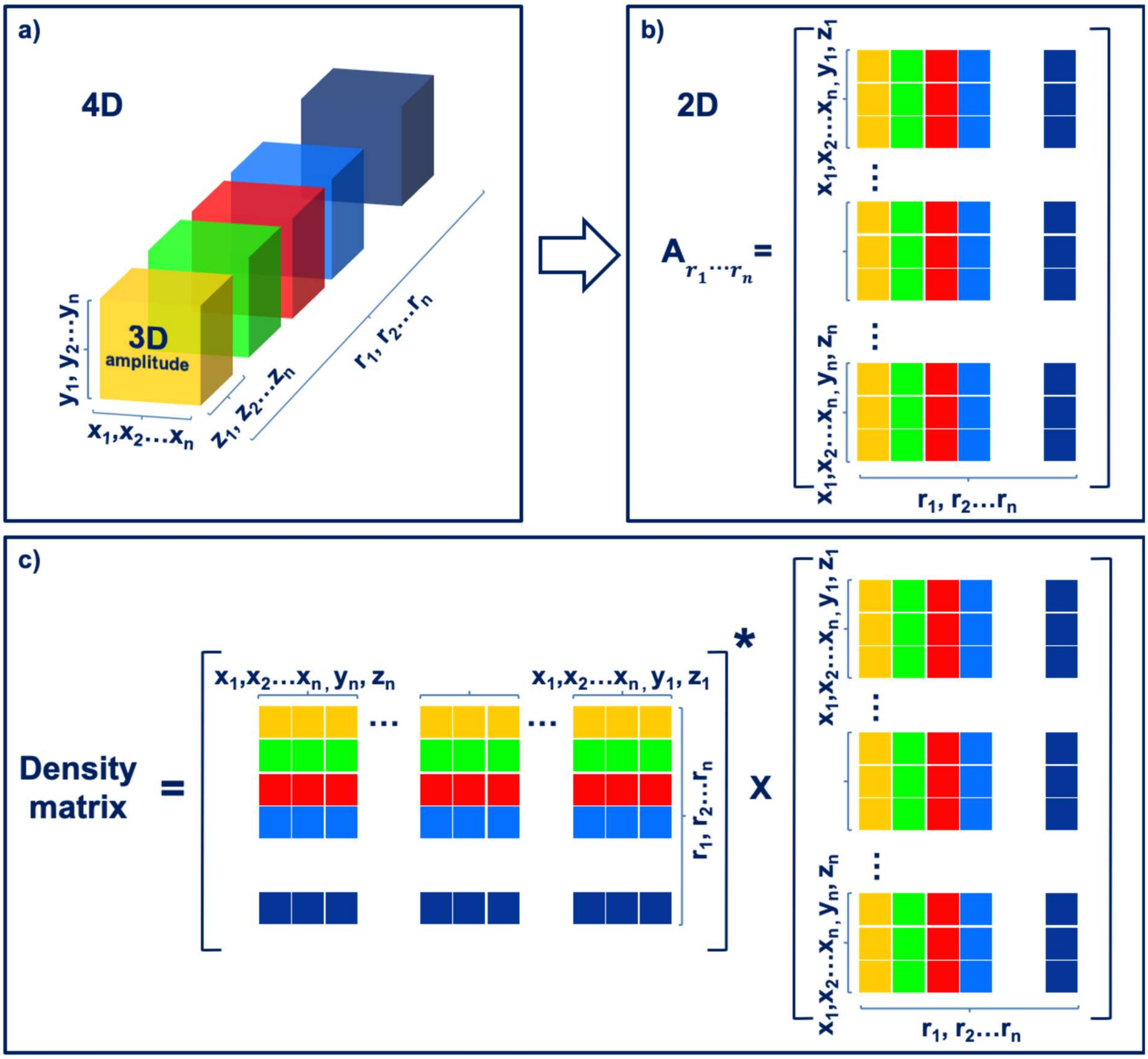

Figure S12 Mode decomposition procedure for the set of the reconstructions obtained by phase retrieval. (a) Initial 4D matrix consisting of 3D amplitudes of the reconstructions (203×203×203 pixels), where the fourth dimension is the number of reconstructions. (b) 4D matrix rearranged to 2D matrix, where each 3D amplitude matrix was rearranged to 1D column, the number of columns corresponds to the number of reconstructions. (c) Density matrix obtained by the multiplication of 2D matrices (b): its transposed complex conjugated and matrix itself.

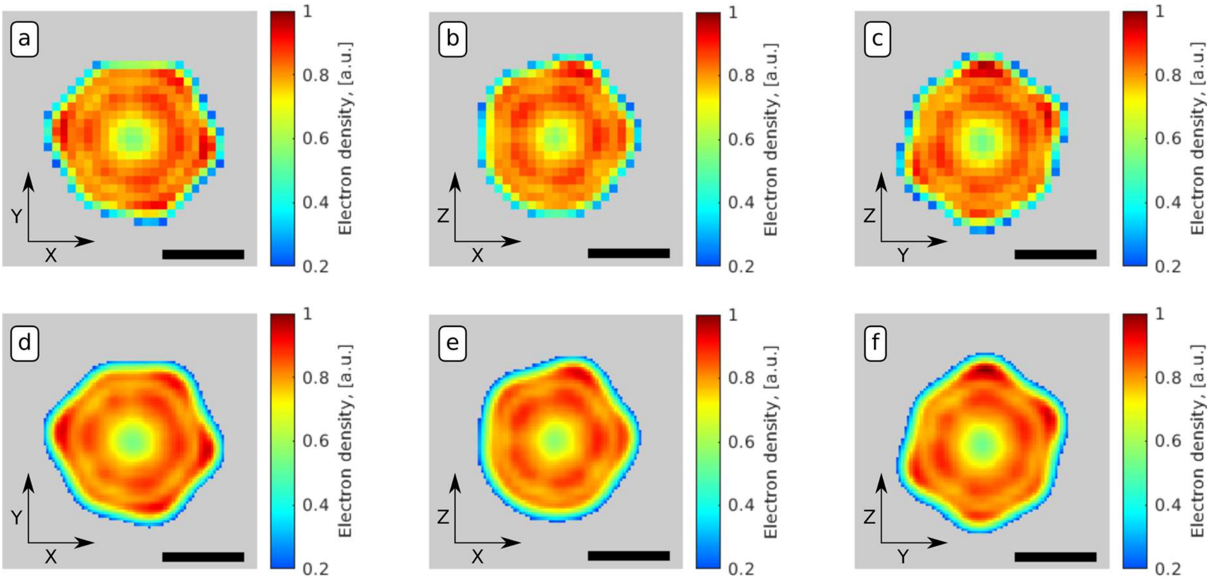

Figure S13 Final electron density of the virus obtained as a result of mode decomposition. Black line denotes 30 nm. (a-c) Results of the initial reconstruction. (d-f) Three times up-sampled results from (a-c). Electron density less than 0.2 was set to grey color scale.

The virus size was obtained from the analysis of the electron density profiles. For the particle size estimate we selected the electron density threshold value of 0.2 as it was considered in the shrink-wrap algorithm during the phase retrieval. From this criterion we determined the particle sizes in the directions from facet to facet and from vertex to vertex (Rose *et al.*, 2018), which are shown in Table S1. The mean particle size was 61 ± 2 nm (between facets) and 63 ± 2 nm (between vertexes).

Table S1 The virus sizes in the directions from facet to facet and from vertex to vertex. The mean sizes in each direction are shown in the last column.

	Size in different directions										Mean size
Facet-to-Facet, (nm)	62±2	60±2	64±2	62±2	60±2	60±2	60±2	59±2	64±2	63±2	61±2
Vertex-to-Vertex, (nm)	63±2	64±2	67±2	61±2	60±2	61±2					63±2

To estimate the capsid size, we analyzed a few electron density profiles (see main text Fig. 6(a-b) and Fig. S14), which were fitted with four Gaussian functions. The area of fitting was considered according to the electron density threshold 0.2, similar to the shrink-wrap value during the reconstruction. Fitting the result for the electron density profile is shown in Fig. S14. Left and right

Gaussian functions correspond to the capsid part of the virus structure. Taking the FWHM values of these curves we determined the capsid size to be 7.6 ± 0.3 nm.

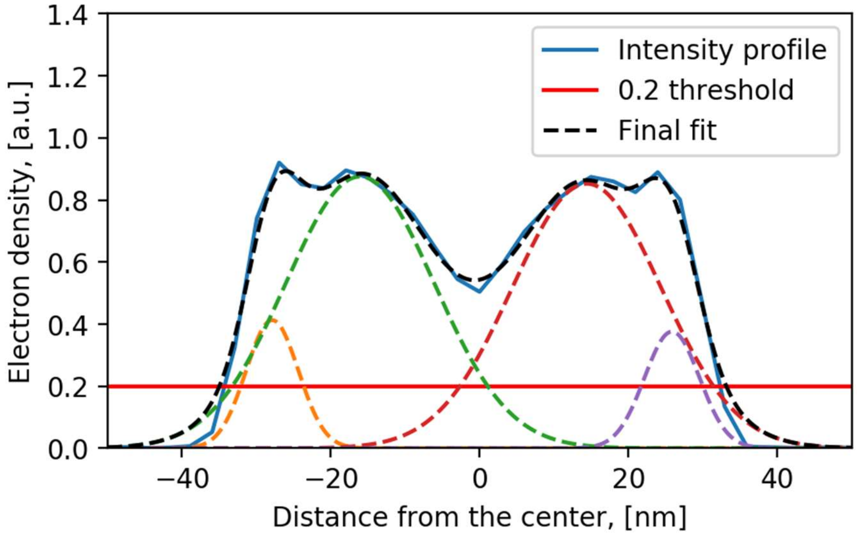

Figure S14 Analysis of the electron density profile. For the capsid size estimate fitting of the electron density line profile with four Gaussian functions was performed. Left and right (orange and purple) Gaussian functions correspond to the capsid. The mean size of the capsid was determined as FWHM of these Gaussian functions and is equal to 7.6 ± 0.3 nm.
